# Pseudohallucination and Pilocytic Astrocytoma in the Pons

**DOI:** 10.1002/brb3.71367

**Published:** 2026-06-25

**Authors:** Ali Mohammadimoshganbar, Bernd Krämer, Memetkuly Kadyrov, Walter Brogiolo

**Affiliations:** ^1^ Cantons Hospital Schaffhausen Schaffhausen Switzerland; ^2^ Psychiatrie Clinik Breitenau Schaffhausen Switzerland; ^3^ Medical University of Zurich Zürich Switzerland; ^4^ Medical University of Aachen Aachen Germany

**Keywords:** astrocytoma, behavioral disorders, brain tumor, fear, hallucination, pons, pseudohallucination, schizophrenia

## Abstract

**Background:**

The pons, a critical structure located in the brainstem, plays a multifaceted role in various neurological functions, and any disruption to its integrity can lead to profound and diverse clinical manifestations. Recent reports have highlighted the association between pontine lesions and conditions such as emotional incontinence, rage, auditory hallucinations, and visual disturbances. We refer to a novel case that causes false hallucinations. We discuss a patient with a tumor in the pons exhibiting a rare constellation of symptoms that included unstable auditory, visual, and perceptual hallucinations. Notably, the patient demonstrated insight into the illusory nature of these perceptions, recognizing that what they experienced was not grounded in reality, yet felt ensnared by these hallucinations, which significantly influenced their behavior and cognitive functioning. Importantly, following the identification and subsequent excision of the pontine tumor, a marked alleviation of the patient's symptoms was observed. This case not only contributes to the limited literature regarding pontine pathologies but also underscores the critical association between pontine integrity and the regulation of sensory and emotional experiences, urging further exploration within the neuroscientific community.

**Case Presentation:**

The patient in this study presented a complex case, initially diagnosed with a schizophrenic episode at a different treatment center, and subsequently referred to our hospital. Upon evaluation, the individual exhibited a range of auditory, visual, tactile, and perceptual hallucinations. What distinguished this case was the patient's intermittent awareness of the unreal nature of these perceptual distortions, which contradicted the typical presentation of schizophrenia, where patients often lack insight into their condition. As we conducted a deeper investigation, it became evident that this patient was actually suffering from a tumor located in the pons area of the brain. This discovery suggested that the psychological disturbances observed were secondary to the tumor's presence rather than a primary psychiatric disorder. The rarity of reports on false hallucinations in similar contexts prompts us to share this case with our colleagues through this article. We aim to highlight the critical role of the brainstem, particularly the pons, in influencing such atypical manifestations of hallucinations. Further research is warranted to explore the underlying mechanisms by which brainstem tumors can lead to these unusual presentations in patients showing symptoms of schizophrenia.

**Conclusion:**

The mere presence of symptoms such as hallucinations should not immediately lead us to a definitive diagnosis of psychological disorders. It is essential to distinguish between different types of hallucinations and consider that psychological disorders like schizophrenia may be secondary symptoms of an underlying primary condition. Without addressing the root cause of the primary illness, these psychological disorder symptoms cannot be effectively treated.

## Graphical abstract

In this graphic, we have briefly shown the process of diagnosing the root cause of the patient's psychiatric and psychological disorders and his treatment. The main reason for strengthening the idea that the primary and root cause of the patient's psychological abnormalities was not schizophrenia was that the patient himself occasionally knew that his hallucinations were not real. With the discovery of the pilocytic astrocytoma tumor and its removal with surgery and proton therapy, the patient's psychological symptoms disappeared.

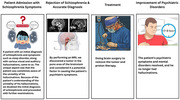

## 1 Introduction

The interplay between neurological disorders and psychiatric manifestations has been a subject of extensive research, providing crucial insights into how physical anomalies within the brain can elicit symptoms that mimic or overlap with established psychiatric diagnoses, such as schizophrenia. Schizophrenia, a chronic and severe mental health disorder, is characterized by a range of symptoms, including delusions, hallucinations, disorganized thinking, and cognitive impairments. While the etiology of schizophrenia has long been considered primarily psychological and neurochemical, emerging evidence now suggests that organic brain lesions may also play a significant role in the emergence of symptoms that resemble those seen in schizophrenia. This report focuses on a singular case of a patient who was misdiagnosed with schizophrenia yet was ultimately found to have a tumor localized in the pons of the brainstem, leading to the manifestation of pseudoschizophrenic symptoms.

The pons is an essential structure of the brainstem, serving as a conduit for signals between different parts of the nervous system. It plays a critical role in various neurological functions, including regulation of sleep, respiration, swallowing, bladder control, hearing, equilibrium, taste, eye movement, facial expressions, and posture (Stopford 1916; Dick et al. 2009; Ioos et al. 2001). Tumors in this region are rare yet can lead to significant dysfunction due to their strategic location. The symptoms arising from pontine lesions may often be mistaken for psychiatric disorders because of their overlap with affective and cognitive disruptions typically seen in conditions such as schizophrenia. This misclassification is particularly concerning, as it can lead to inappropriate treatment regimens that do not address the underlying medical condition, potentially exacerbating the patient's suffering.^1^

A noteworthy observation in this case involves the nature of the hallucinations experienced by the patient. Unlike classic auditory or visual hallucinations associated with schizophrenia, the patient sometimes demonstrated awareness that his hallucinations were not grounded in reality, a crucial distinction often overlooked in psychiatric evaluations. This phenomenon presents an intriguing divergence from traditional psychiatric presentations and raises important questions regarding the criteria used to diagnose and understand psychotic disorders. The discrepancy in the patient's ability to discern the unreal nature of his experiences adds a layer of complexity that warrants attention. It illuminates the crucial need for comprehensive diagnostic assessments that encompass not just psychiatric evaluations but also thorough neurological examinations, particularly in cases where symptoms are atypical or do not conform neatly to established diagnostic frameworks.

The uniqueness of this case underlines significant implications for both clinical practice and theoretical frameworks concerning the relationship between organic brain pathology and psychiatric symptoms. It serves as a potent reminder of the intricate relationship between mental and physical health, emphasizing that the presentation of psychiatric symptoms may often stem from underlying neurological conditions that require a different therapeutic approach. Moreover, it stresses the importance of a multidisciplinary approach in the assessment and treatment of such patients, ensuring that neurological evaluations are prioritized alongside psychiatric assessments when presented with ambiguous or atypical symptoms.

The aim of this report is to shed light on the interplay between a pontine tumor and the emergence of symptoms that superficially resemble schizophrenia, thereby contributing to the growing body of literature advocating for a more nuanced understanding of psychosis in the context of brain pathology.

## 2 Case Presentation

A 25‐year‐old male with autism spectrum disorder (F84: the patient has challenges in social interaction, significant difficulties in communication, and restrictive and repetitive patterns of behavior) presented to our center following a diagnosis of paranoid schizophrenia (F20) at another hospital, where he had been prescribed medication.

The diagnosis of schizophrenia at the primary center was based on the patient's following symptoms:
*Psychiatric symptoms*:
He experienced multimodal hallucinations:

- Auditory: indistinct voices, noises, sound distortions
- Visual: fleeting figures, distortions in shapes and colors
- Tactile and visceral sensations: abnormal heat sensations, “pressure,” crawling feelings
- Hypnagogic intrusions: vivid dreams blending into wakefulness.

Crucially, insight fluctuated; for example, he attempted to “fly” after dreaming he had supernatural powers, but simultaneously acknowledged that “in real life” he could not fly. This preserved insight suggested pseudohallucinations, which are atypical for schizophrenia.
*Behavioral and emotional symptoms*:

- Marked anxiety and agitation, especially at night
- Tearfulness and emotional dependency
- Fear of being alone
- Sleep fragmentation with nightmares
- Reduced appetite and fluid intake

*Neurological and autonomic symptoms*:

- (All documented in the original PDF and reorganized here)
- Breathing difficulty
- Dysphagia
- Gait instability with falls
- Sensation of heavy legs
- Episodic temperature elevation (37.1°C–39.6°C)
- Perceptual distortions regarding body size
- Severe fatigue

In our opinion, these symptoms significantly diverged from typical schizophrenia and prompted further neurological evaluation.

We present this case to illustrate the patient's symptomatology and our rationale for ultimately rejecting the schizophrenia diagnosis, despite the presence of delusions and hallucinations. We describe our diagnostic process and the successful treatment of the patient's delusions, hallucinations, and altered mental state.

We describe some of the patient's symptoms in more detail:

*Hallucinatory Experiences, Psychotic Symptoms, and Severe Sleep Disturbance*

The patient exhibited episodes of perceptual disturbances that caused significant emotional distress, intermittent psychotic features, and notable impairment of sleep. Examples include:

*Example A*: The patient reported vivid dream‐like experiences in which he perceived himself as a supernatural being with abilities such as flying, telepathy, and extraordinary strength. On one occasion, while awake, he stood on a balcony with his arms outstretched, stating that he intended to fly. He explained that he had dreamt of himself flying the night before. Importantly, he demonstrated partial insight, acknowledging at times that he could not actually fly in reality. This partial awareness indicated the presence of pseudohallucinations rather than fully formed true hallucinations. Although he experienced vivid sensory phenomena and briefly acted in response to them, his intermittent recognition of their unreal nature suggested an underlying psychological process, prompting further diagnostic exploration.

*Example B*: The patient believed that his ring was the source of his back pain and perceived it as threatening. After the ring was removed, he reported immediate relief and expressed that the perceived threat had resolved, which coincided with clinical improvement.

*Example C*: The patient frequently experienced heightened anxiety and episodes of crying, associated with false hallucinatory perceptions involving auditory, tactile, visual, and somatic sensations.

*Affective Symptoms and Insight*

The patient frequently expressed anger toward himself. Although he demonstrated intermittent insight by recognizing at times that his perceptual disturbances were not real, he was unable to understand the origin of these experiences, which contributed to significant frustration. Despite these affective symptoms, there were *no indications of self‐harm, suicidality, or risk to others*.

*Changes in Personality, Increased Dependency, and Need for Support*

The patient's father reported a notable change in his son's personality, characterized by an atypical longing for his parents and increased emotional dependency. In addition, the director of the residential institution—where the patient has resided on weekdays since November 2021—observed that the patient increasingly sought contact and conversation. This behavior appears to reflect heightened fear of being alone and an increased need for reassurance, likely triggered by distressing pseudohallucinatory experiences (e.g., hearing unclear or indistinct voices). The patient found these experiences profoundly unsettling and was unable to cope with them independently, resulting in a marked increase in his need for emotional support and interpersonal interaction.

*Night‐Time Agitation and Sleep Disturbance*

The patient reported significant nocturnal agitation, tension, and anxiety. He experiences frequent nightmares, which prevent restorative sleep and result in daytime fatigue. His sleep pattern is further disrupted by consistently going to bed late and awakening early.

*Reduced Appetite and Fluid Intake*

The patient's appetite and overall fluid consumption have decreased, contributing to concerns about his general physical well‐being.

*Respiratory Difficulties*

The patient exhibits symptoms suggestive of respiratory discomfort or difficulty, requiring further medical evaluation to determine the underlying cause.

*Dysphagia*

The patient reports difficulty swallowing, which may be related to anxiety, somatic preoccupation, or a concurrent medical condition. Further assessment is warranted.

*Motor Symptoms, Somatic Perceptual Distortions, and Balance Issues*

The patient reports impaired balance resulting in falls. He describes his legs as feeling unusually heavy and appearing larger than normal, and perceives his head as disproportionately large. These somatic distortions may reflect *body image misperceptions*, anxiety‐related somatic symptoms, or disturbances in proprioception and require further neurological and psychiatric evaluation.

*Fluctuating Body Temperature*

The patient demonstrated intermittent elevations in body temperature, with documented fluctuations ranging from 37.1°C to 39.6°C. These periodic changes may indicate an underlying infection, autonomic dysregulation, or stress‐related physiological responses. The pattern warrants further medical evaluation to exclude organic causes, including infectious, inflammatory, or endocrine processes.

As described, the above‐mentioned patient, exhibiting symptoms such as hallucinations, delusions, and severe mental disorders, had visited another hospital where he was diagnosed with schizophrenia. The following medications were prescribed for him.

− Lorazepam 1 mg, 1⁄2–1⁄2–0–1⁄2
− Risperidon 1 mg/mL, 100 mL, 1–0–0–2 mg
− Redormin 250 film‐coated with the active ingredients: valerian root, hop cone extract

2 pieces, max. 6 pieces/24 h

Later, the patient visited our hospital and was examined and evaluated in our psychiatric department. We concluded that there might be another cause for these psychiatric issues due to the pseudodelusions and pseudohallucinations, since in psychosis and schizophrenia, the patient typically perceives hallucinations as reality. However, this patient occasionally recognized the hallucinations as unreal.

In summary, some of our reasons for rejecting schizophrenia as the primary cause of symptoms are as follows:

Preserved insight is atypical in schizophrenia.
DSM‐5 and ICD‐10 require impaired reality testing for psychotic disorders. This patient repeatedly recognized that some experiences “were not real,” conflicting with a schizophrenia diagnosis.
Neurological red flags must not be overlooked.
Dysphagia, gait disturbance, autonomic instability, and sensory distortions cannot be explained by schizophrenia and should prompt neuroimaging.
Pontine lesions can mimic psychosis.
Prior literature documents that brainstem lesions can cause hallucinosis, emotional dysregulation, and impulse disturbances. This patient's symptoms were fully consistent with known effects of pontine dysfunction.
Rapid postoperative improvement supports organic etiology.
The immediate disappearance of hallucinations within 48 h is incompatible with schizophrenia, which does not resolve acutely after neurosurgical intervention.

In patients with schizophrenia‐like symptoms, certain atypical or concerning clinical features should raise suspicion for an underlying organic pathology and prompt further investigation, including MRI. Red flags include:

Autonomic dysfunction (e.g., orthostatic hypotension, fever, labile blood pressure, abnormal sweating), suggesting brainstem or systemic involvement.
Rapid or progressive cognitive decline, especially in young patients—disproportionate memory loss, aphasia, or marked executive impairment.
Focal neurological deficits (e.g., hemiparesis, sensory loss, visual field defects, dysphasia), indicating potential structural lesions.
Seizures, particularly focal or new‐onset, raising concern for epilepsy or intracranial pathology.
New‐onset psychosis in middle age or later, which may reflect dementia, stroke, tumors, or CNS infections.
Abrupt or rapidly evolving psychotic symptoms, suggestive of delirium, intoxication, metabolic causes, or encephalitis.
Hallucinations accompanied by other neurological signs (e.g., visual hallucinations with confusion or motor changes), pointing to occipital/temporal lobe pathology or inflammatory processes.
New abnormal movements (tremor, dystonia, myoclonus, parkinsonism), which may indicate neurodegeneration, metabolic disease, or infection.
Systemic illness or signs of infection, particularly fever or meningism, raising concern for encephalitis or meningitis.
Severe or changing headaches, especially with vomiting or focal deficits, suggestive of space‐occupying lesions or raised intracranial pressure.
These features warrant prompt medical evaluation to exclude organic causes that may mimic or complicate schizophrenia.

Further examinations and an MRI revealed that the patient had pilocytic astrocytoma in the Pons region with the following characteristics:

Partially cystic, partially solid, and markedly inhomogeneous contrast‐enhancing intra‐axial tumor located paramedian to the right in the pons. Dimensions approximately 3.5 cm × 3.3 cm × 2.6 cm. Rather mild perilesional edema, no significant narrowing of cerebrospinal fluid spaces yet, no hydrocephalus. Suspected pons glioma.

Through further examinations and an MRI (above images), we reached the following diagnosis: Pilocytic astrocytoma (WHO Grade 1) in the pons.1 2

Below are the results of the tumor's immunohistochemical tests:

Positive with GFAP and H3 K27me.

**FIGURE 1:**
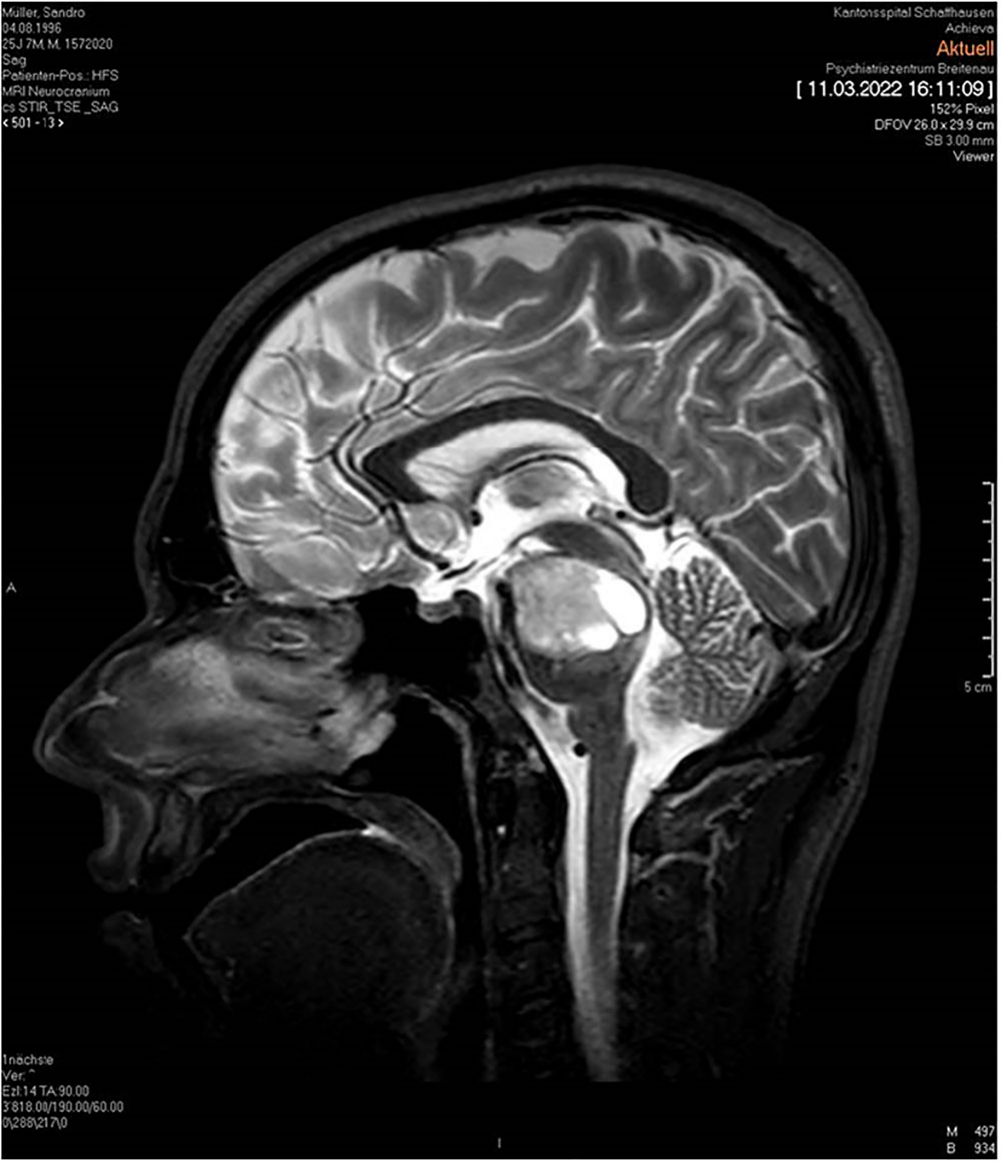
Brain MRI. T2w STIR sagittal: Partially cystic tumor in the significantly swollen pons (the “brightest” parts are the cysts).

**FIGURE 2:**
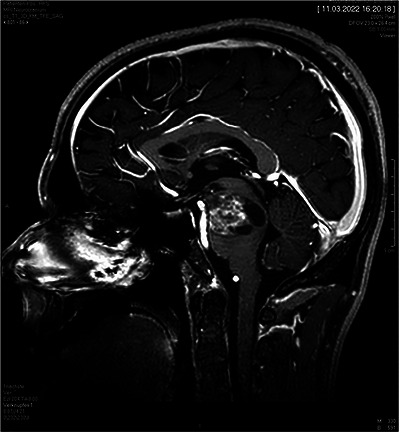
MRI findings are illustrated. After IV contrast administration, the non‐cystic parts in the T1w 3D fs sagittal show an inhomogeneous contrast enhancement (“bright spots”).

We considered the aforementioned tumor as a potential cause for the patient's psychological disorders. The 40%–50% of the tumor was removed and the patient experienced significant changes, as outlined below:

*Postoperative clinical course*:

During 0–48 h after surgery:

- Complete resolution of auditory and visual hallucinations within 48 h
- Marked reduction in fear, anxiety, and agitation
- Sleep improved the first night with fewer awakenings
- Temperature normalized

First week

- Tactile hallucinations disappeared by postoperative Day 4
- Emotional stability improved; patient no longer sought continuous reassurance
- Gait and balance improved significantly with physiotherapy
- Appetite normalized

Weeks 2–4

- Mild residual anxiety gradually resolved
- Normal sleep architecture returned
- Social interaction improved; no dependency behaviors

Week 6

- No hallucinations, delusions, agitation, or fear
- Returned to baseline functioning for ASD
- No recurrence of neurological symptoms

## 3 Discussion

In exploring the relationship between pontine tumors and the manifestation of hallucinations (Papantoniou et al. 2023), particularly the unique category of “pseudohallucinations,” we have identified significant clinical implications that can inform both diagnosis and treatment. Pseudohallucinations, which are characterized by the individual's awareness that their perceptions are not grounded in reality, starkly contrast with the genuine hallucinations often encountered in schizophrenia, marked by a lack of such insight. By distinguishing between these types of hallucinations, we can move toward a more nuanced understanding of the underlying causes and effective treatments for the patient.

Upon confirming the presence of a tumor in the pons region and its resultant interference with brainstem function, the patient exhibited symptoms consistent with prior medical literature. Notably, the pons is associated with emotional dysregulation and aggressive behaviors (Kim and Choi‐Kwon 2000; Kim et al. 2002). Previous reports indicate that pontine lesions may include auditory and visual hallucinations (Lee et al. 2008; Cascino and Adams 1986), as well as disturbances in affective regulation. Furthermore, unverified reports suggest a correlation between pontine infarction and increased irritability, impulsivity, and dysphoric behaviors, in addition to complex behaviors such as delusions of infidelity (Kim et al. 1994). The alignment of our patient's symptoms with these documented findings created a compelling rationale to investigate whether treatment of the pons tumor would markedly alleviate the psychiatric symptoms.

Following the surgical removal of the tumor from the pons, we observed a significant improvement in the patient's psychological distortions. This outcome strongly suggests that the psychological disturbances of our patient were not indicative of a primary psychiatric disorder such as schizophrenia. Instead, they were secondary effects attributable to the tumor's impact on neurobiological pathways. Thus, the tumor appeared to be a substantial contributing factor to the patient's aberrant cognitive experiences.

But why do organic psychiatric disorders often present with pseudohallucination and preserved insight, compared to primary schizophrenia?

Organic disorders often produce pseudohallucinations and preserved insight because the hallucinations are typically linked to identifiable brain dysfunction or medical conditions, and patients maintain some level of cognitive ability to recognize the abnormality of their perceptions.

In contrast, primary schizophrenia involves chronic neurodevelopmental changes that can profoundly disrupt cognitive function and perception, leading to true hallucinations and poor insight.

Understanding these differences is crucial for diagnosing and distinguishing between organic psychiatric disorders and primary schizophrenia, as treatment approaches and prognoses can vary significantly.

An essential question arises: *Had we misinterpreted the patient's pseudohallucinations as true psychotic phenomena and failed to pursue further medical evaluation, would neuroimaging have ever been performed?* If the patient had lacked insight and fully believed his perceptual disturbances to be real—or if clinicians had prematurely labeled the symptoms as schizophrenia, as occurred in the previous treatment setting—the underlying pontine lesion may have remained undiagnosed. Such a scenario would likely have resulted in continued clinical deterioration and placed the patient at significant risk.

This case therefore underscores a critical principle: *patients presenting with complex, atypical, or fluctuating psychiatric symptoms should undergo thorough diagnostic assessment, including neuroimaging, to evaluate for possible central nervous system pathology*. Brain lesions, even when rare, can mimic or exacerbate psychiatric syndromes and must remain part of the differential diagnosis.

However, implementing routine MRI screening for all patients with psychosis presents practical challenges. Financial constraints, limited access to imaging, and the difficulties of performing MRI scans in acutely disturbed psychiatric patients require thoughtful deliberation. Interdisciplinary communication—particularly between psychiatry, neurology, and radiology—is essential to balance clinical necessity with resource allocation and patient safety.

To improve diagnostic accuracy and prevent future misdiagnoses, *the development of clearer clinical protocols is warranted*. We recommend:

- *Systematic research* investigating the prevalence of structural brain lesions in patients diagnosed with schizophrenia or schizophrenia‐like disorders.
- *Retrospective and prospective studies* to evaluate whether cases of atypical psychosis may have included unrecognized neurological etiologies.
- *Standardized screening guidelines* outlining when neuroimaging should be strongly considered, particularly in the presence of atypical features such as preserved insight, neurological deficits, autonomic instability, or abrupt symptom onset.

Such research would allow the field to determine whether certain patients previously labeled as having primary psychiatric illnesses may, in fact, have had misdiagnosed or overlooked neurological conditions. This, in turn, would enable the reporting of prevalence data, strengthen clinical awareness, and ultimately enhance treatment quality and patient outcomes.

In conclusion, this case highlights the indispensable role of comprehensive evaluation in psychiatry and supports the integration of neuroimaging into the assessment of patients with complex or atypical psychotic symptoms.

## 4 Conclusion

This case demonstrates that the patient's previously assigned diagnosis of a schizophrenic episode was likely inaccurate. Although the patient presented with hallucinations and delusional thinking, his preserved insight—specifically his ability to recognize that these experiences were not real—was inconsistent with a primary psychotic disorder. Comprehensive diagnostic evaluation, including MRI, revealed a tumor in the pons. Based on current medical literature and observed clinical presentation, this pontine lesion provided a coherent organic explanation for the patient's psychiatric symptoms.

Following neurosurgical removal of the tumor, the patient's hallucinations, delusions, and associated behavioral disturbances resolved rapidly and completely. This dramatic improvement strongly supports the conclusion that the psychiatric symptoms were secondary to brainstem dysfunction rather than indicative of schizophrenia. The case highlights a rare and underreported phenomenon in which pontine pathology produces complex perceptual disturbances resembling pseudohallucinations or psychosis.

This experience underscores the necessity for clinicians to maintain a high level of vigilance when evaluating patients with atypical or incomplete psychotic presentations. Symptoms such as preserved insight, neurological deficits, autonomic instability, dysphagia, gait disturbance, or unusual somatic perceptions should prompt immediate consideration of an organic etiology, including structural lesions of the brainstem. Brain imaging—despite cost, resource limitations, or logistical challenges—may be essential in avoiding misdiagnosis and preventing life‐threatening delays in treatment.

There is a clear need for more precise clinical documentation and scientific investigation into how dysfunction of the brainstem, particularly the pons, can generate psychiatric symptoms traditionally associated with primary psychotic disorders. Incorporating systematic neurological assessment and neuroimaging into diagnostic pathways for patients with schizophrenia‐like symptoms has the potential to significantly improve diagnostic accuracy, guide appropriate treatment, and ultimately enhance patient outcomes and quality of life.

This case reinforces a critical principle in psychiatry and neurology: the presence of psychotic symptoms should never preclude a thorough medical evaluation, and structural brain lesions should always remain on the differential diagnosis when atypical features are present.

## Author Contributions

**Ali Mohammadimoshganbar**: writing – review and editing, writing – original draft, conceptualization, methodology, software, data curation, resources, formal analysis, project administration, validation, visualization, investigation, and supervision. **Bernd Krämer**: writing – review and editing, supervision, formal analysis, validation, and investigation. **Memetkuly Kadyrov**: writing – review and editing, methodology, resources, validation, formal analysis, and supervision. **Walter Brogiolo**: conceptualization, investigation, writing – review and editing, visualization, validation, methodology, formal analysis, project administration, resources, supervision, and data curation.

## Funding

The authors have nothing to report.

## Data Availability

The data that support the findings of this study are available from the corresponding author upon reasonable request.

## Endnotes

1 In this article, “pseudo and false hallucination” refers to when an individual knows their perceptions are not real and can separate them from reality. While there are categories for real and false hallucination, no precise classification exist for the different types based on their causes (El‐Mallakh and Walker 2010; Berrios and Dening 1996)
